# Rewritable Painting Realized from Ambient-Sensitive Fluorescence of ZnO Nanoparticles

**DOI:** 10.1038/srep42232

**Published:** 2017-02-07

**Authors:** Kai-Kai Liu, Chong-Xin Shan, Gao-Hang He, Ruo-Qiu Wang, Lin Dong, De-Zhen Shen

**Affiliations:** 1 State Key Laboratory of Luminescence and Applications, Changchun Institute of Optics, Fine Mechanics and Physics, Chinese Academy of Sciences, Changchun 130033, China; 2 University of Chinese Academy of Sciences, Beijing 100049, China; 3 School of Physics and Engineering, Zhengzhou University, Zhengzhou 450001, China

## Abstract

Paper, as one of the most important information carriers, has contributed to the development and transmission of human civilization greatly. Meanwhile, a serious problem of environmental sustainable development caused by the production and utilization of paper has been resulted to modern society. Therefore, a simple and green route is urgently demanded to realize rewritable painting on paper. Herein, a simple route to rewritable painting on copy paper has been demonstrated by using eco-friendly ZnO nanoparticles (NPs) as fluorescent ink, and vinegar and soda that are frequently used in kitchen as erasing and neutralizing agents. Words or patterns written using the ZnO NPs as ink can be erased by vinegar vapour within five seconds, and after a neutralizing process in the ambient of soda vapour, the paper can be used for writing again. It is worth noting that the resolution and precision of the patterns produced via the above route degrade little after ten rewriting cycles, and the quality of the patterns produced using the ZnO NPs as ink fades little after being storage for several months, which promises the versatile potential applications of the rewriting route proposed in this paper.

Writing is one of the most common activities of human society, and most of the writing is carried out on paper, and paper can usually be used only once, which leads to the huge consumption of paper every day. Much wood is thus needed to supply the extensive use of paper, which cause severe deforestation^1^. Additionally, the disposed paper creates quite a few solid wastes and serious chemical pollution to air, water and land^2^. It is natural to speculate that if a piece of paper can be used for multiple times, the cost for consumers and the contamination to environment will be reduced greatly. In fact, multifarious dyes with reversible colour switching based on external-stimuli as imaging layers prevails in rewritable area^3^,^4^,^5^,^6^,^7^,^8^ and substantial progress has been made in this vital area in recent years^9^,^10^,^11^,^12^,^13^,^14^. For instance, Sheng *et al*. demonstrated a rewritable paper, in which the paper incorporated with switchable dyes as imaging layer and water as ink. Because of the hydrochromic properties of the dyes, when water is printed onto the dye incorporated paper, words or patterns will be produced^15^. Wang *et al*. have demonstrated a rewritable paper based on color switching of redox dyes by using titanium dioxide (TiO_2_) assisted photocatalytic reactions. In this route, a solid film composed of TiO_2_ nanocrystals, a redox dye, and hydroxyethyl cellulose, is employed as “paper”, and ultraviolet (UV) light that can cause the photocatalystic reaction of TiO_2_ as a “pen”. Under UV illumination, photocatalystic activity of the TiO_2_ will cause the reduction of the dyes thus the color of the dyes changes, in this way, words can be written. On heating in oxygen ambient, the reduced dyes will be oxidized, thus the color of the dye will be recovered^16^. It can be seen that the considerably complex preparation procedure or modification to the paper must be involved in the above cases, which will result in a significant increase in the cost of paper. Furthermore, the words and patterns produced via the above processes are sensitive to light or humidity, which is unfavorable for their long-term storage. If a rewritable route is developed on common copy paper in a simple way, the significance of such route will be self-evident.

In this paper, by using ZnO NP aqueous solution as fluorescent ink, from which strong fluorescence can be realized under UV illumination, a simple route has been demonstrated to rewritable painting on paper. The fluorescence of the ZnO NPs can be quenched greatly by acid^17^,^18^,^19^, so the written words or patterns can be erased by placing the paper in the ambient of vinegar vapour. For rewriting, the vinegar treated paper needs to be neutralized in soda vapour, then the paper can be used for writing or painting again. In this way, a rewritable route has been realized, and the precision and resolution of the words or patterns produced in this route degrade little after tens of cycles, indicating its good rewritten ability. Note that the agents like vinegar and soda used in this study have been widely used in kitchen, and ZnO has also been highlighted for its eco-friendliness and low cost^20^,^21^,^22^,^23^,^24^,^25^,^26^. Therefore, the results reported in this paper may provide a simple and green route to rewritable painting on paper, thus will be of great significance in saving the usage of paper, thus help to reduce the vast pollution to air, land, and water caused during the making, recycling, and disposal of paper.

## Results

**Figure 1:**
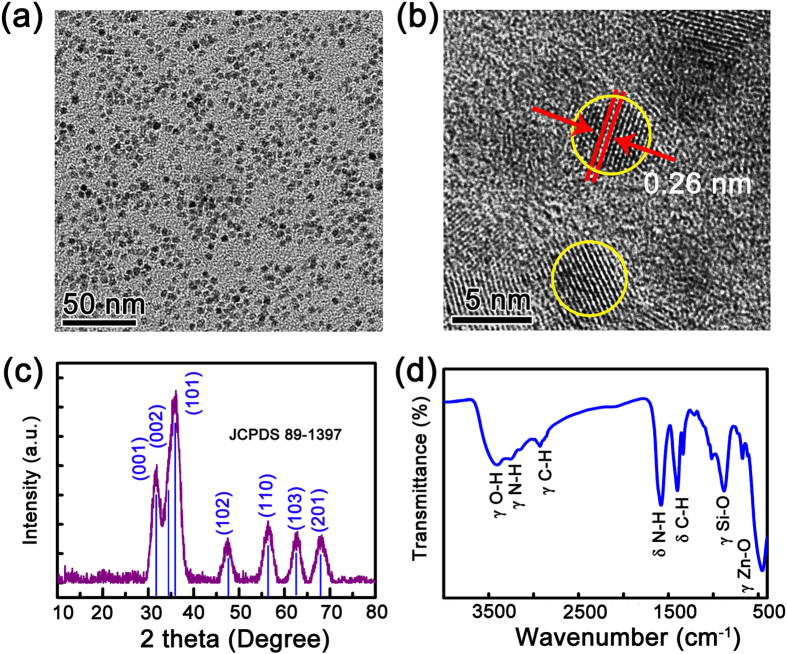
(**a**) TEM image of the ZnO NPs; (**b**) High-resolution TEM image of the NPs; (**c**) XRD pattern of the ZnO NPs; (**d**) FTIR spectrum of the ZnO NPs.

The morphology and structural characterizations of the ZnO NPs employed as the fluorescent ink in this communication are shown in Fig. 1(a). It is indicated that the NPs are spherical in shape with a diameter of 3–5 nm. Clear lattice fringes of the NPs (marked in yellow circle) with a spacing of about 0.26 nm can be observed in the high-resolution transmission electron microscope (HRTEM) image, confirming the well-crystalline nature of the NPs, as shown in Fig. 1(b). X-ray diffraction (XRD) is used to characterize the crystalline properties of the NPs further, and some broad diffraction peaks can be observed from the XRD pattern, as illustrated in Fig. 1(c). All the peaks can be attributed to the diffraction from wurtzite ZnO, and the broadening of the diffraction peaks may be due to the small size of the NPs^27^. Note that to make the NPs compatible well with ink-jet printers as fluorescent ink, water-soluble NPs will be a better choice. Therefore, the NPs have been modified by silane coupling agents^28^. To verify that the ZnO NPs have been modified well, Fourier transform infrared (FTIR) spectra of the ZnO NPs have been recorded by the KBr method, as indicated in Fig. 1(d). The stretching vibration band of Si-O (around 1000 cm^−1^) can be observed clearly, indicating that silane has been coated onto the ZnO NPs^29^. The stretching vibration bands of N-H and O-H (3000–3500 cm^−1^) are also visible in the figure, promising that the NPs can be dispersed well in aqueous solution owing to the existence of these hydrophilic functional groups.

**Figure 2:**
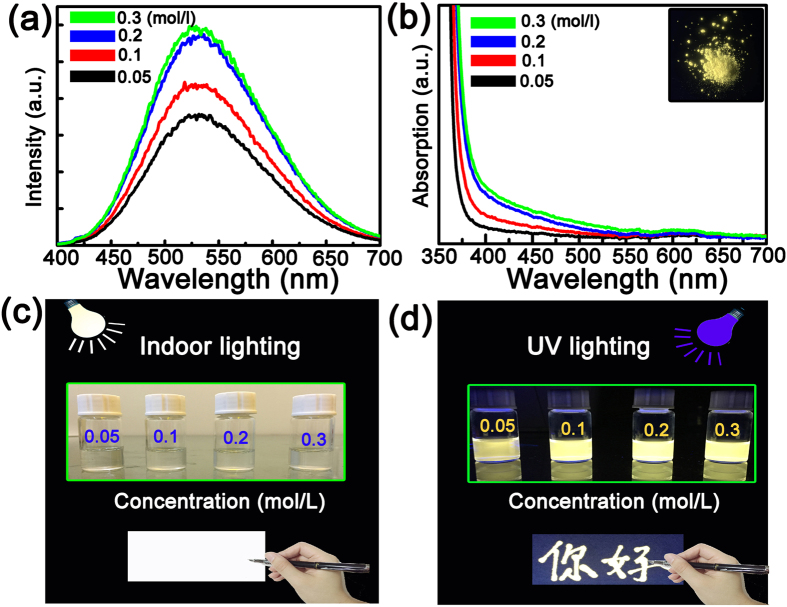
(**a**) The fluorescence spectra of the ZnO NP solution with concentrations ranging from 0.05 to 0.3 mol/L; (**b**) Absorption spectra of the ZnO NPs with different concentrations, and the inset shows the fluorescence image of solid NPs evaporated from the NP solution; (**c**) The images of the NPs and the written words using NP solution as ink under indoor lighting conditions and (**d**) UV lighting conditions. (The photographs of hand was taken by Ruo-Qiu Wang).

To test whether the ZnO NPs can be employed as fluorescent ink, the fluorescence spectra of the NPs have been recorded, as shown in Fig. 2(a). The spectra are dominated by a broad emission at around 525 nm, which is commonly observed in ZnO NPs, and can be attributed to the deep-level emission of ZnO^30^,^31^,^32^,^33^,^34^. The fluorescence intensity increases gradually with its concentration in the investigated range. The absorption spectra of the ZnO NPs with different concentrations are shown in Fig. 2(b). It is evidenced that all the spectra show a strong absorption in the region shorter than 400 nm, while in the visible region the absorption is almost negligible. The inset of Fig. 2(b) is the fluorescence image of solid ZnO NPs evapourated from the aqueous solution under the illumination of the 365 nm line of a mercury lamp, indicating the NPs have a strong fluorescence even in solid conditions, which lays a solid ground for the ZnO NPs being employed as fluorescent ink. Several Chinese characters have been written on fluorescer-free copy paper using the ZnO NP solution with concentration of 0.3 mol/L as ink, as indicated in Fig. 2(c). Since the ZnO solution is colorless and transparent, no word can be observed from the paper under normal indoor lighting conditions, while under UV illumination, the words can be clearly observed due to the fluorescence of the ZnO NPs under UV illumination. The above phenomenon reveals that the ZnO NPs can be employed as fluorescent ink for writing on paper.

**Figure 3:**
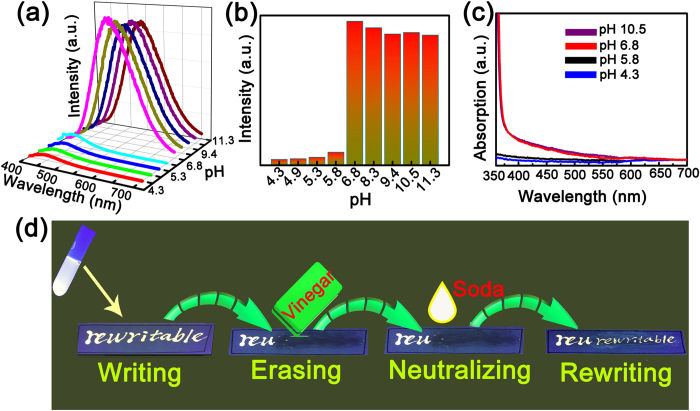
(**a**) The fluorescent spectra of the ZnO NPs under different pH value; (**b**) The histogram diagram of the fluorescence intensity of the ZnO NPs under various pH values; (**c**) Absorption spectra of the ZnO NPs with pH value of 4.3, 5.3, 6.8, and 10.5 respectively; (**d**) The schematic illustration of the writing, erasing, neutralizing, and rewriting process using the ZnO NP solution as ink, vinegar as eraser, and soda solution as neutralizer.

The fluorescence of the ZnO NPs can be quenched greatly in acidic conditions, as shown in Fig. 3(a), note that the pH value is adjusted by dipping several drops of vinegar into the ZnO NPs solution. It is obvious from the figure that the fluorescence intensity of the ZnO NPs decreases greatly in acidic conditions, while it degrades little in alkaline conditions. The above phenomenon can also be clearly observed from the fluorescence intensity histogram of the ZnO NP solution indicated in Fig. 3(b). It is obvious that under UV illumination, the solution is colorless in acidic conditions, while in neutral or alkaline conditions, the solution shows strong brightness, confirming that the fluorescence of ZnO NPs can be quenched in acidic ambient. To investigate the quenching mechanism, the absorption spectra of the ZnO NPs solution with different pH values have been measured, as illustrated in Fig. 3(c). One can see from the figure that the strong absorption shorter than 400 nm has almost disappeared in acidic conditions, revealing that the fluorescence quenching is primarily due to the decomposition of the ZnO NPs in acidic conditions. The fluorescence quenching of the ZnO NPs in acidic ambient indicates that the words written using ZnO NPs as fluorescence ink can be erased effectively by acid. To verify the above speculation, some words have been written on copy paper using the ZnO NPs solution as ink, and the characters can be observed clearly under UV illumination. After placing the paper in the ambient of vinegar, the characters have disappeared, indicating that the written words can really be erased effectively by acid. Furthermore, it is expected that when the surrounding of the NPs is neutralized, the words may be rewritten again. Thus the vinegar contaminated paper has been placed in the ambient of soda vapour to neutralize the absorbed vinegar, words can be written onto the paper again, and the schematic illustration of the above process is shown in Fig. 3(d).

**Figure 4:**
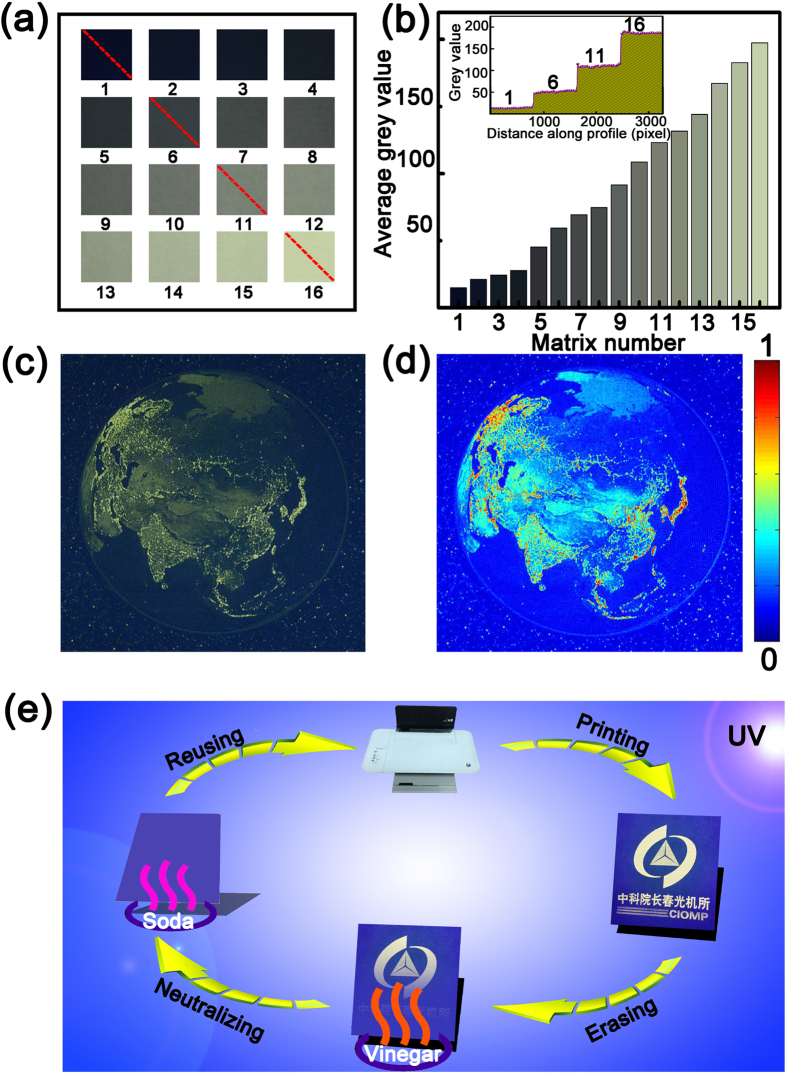
(**a**) A 4 × 4 grey scale pattern printed by using ZnO NP solution as ink; (**b**) The variation of grey value of each square, and the inset is the grey value along the yellow line marked in (**a**); (**c**) The printed night-time image of the earth pictured by NASA; (**d**) Simulated image of (**c**) with MATLAB; (**e**) Schematic illustration of the rewriting process in a printer employing the ZnO NPs as fluorescent ink.

As mentioned above, the handwritten characters using ZnO NPs as fluorescent ink can be erased by vinegar and they can be rewritten again when the acidic paper is neutralized in soda vapour environment, indicating the feasibility of this route of rewriting on paper. For further practicability, it is expected that if more complicated patterns can be rewritten using the above procedure, the significance of such route will be much more great. Nevertheless, to realize more precision and clearness of the patterns using ZnO NPs as ink, multiple channels of grey scale are necessary. To test whether multiple grey scales can be realized, the ZnO NP aqueous solution is filled into the cartridge of an ink-jet printer, and a matrix containing sixteen squares with different grey scales has been printed. One can see that every printed square with width of 2.5 cm has distinctive color density, as indicated in Fig. 4(a). The average grey value of each printed squares has been calculated with MATLAB software and presented in Fig. 4(b). Also, the grey value along the red line in Fig. 4(a) is calculated and the grey value within each square is almost constant, which means the pattern can be printed accurately, as shown in the inset of Fig. 4(b). To verify its practicability, a night-time image of the earth pictured by NASA is printed onto paper employing the ZnO NP aqueous solution as ink, as shown in Fig. 4(c). The image is transferred into pseudo-color image, so it can be described in a quantitative way, as indicated in Fig. 4(d). Based on the above process, it is indicated that the ZnO NP solution can be used as fluorescent ink to print precise patterns. To test whether the paper can be used multiple times for complicated patterns, a pattern has been printed onto a copy paper. The patterns can be observed clearly under UV illumination owing to the fluorescence characteristics of the ZnO NPs. Then, the printed patterns are placed above the vinegar vapour, which results in the fluorescence quenching of the ZnO NPs. Thus the patterns disappear even under the illumination of UV light, which means the printed patterns have been erased. To achieve the reuse of the paper, the paper is placed above the vapour of soda solution to neutralize the absorbed vinegar. At this stage, no pattern is visible from the neutralized paper even under UV illumination. After neutralization, the paper can be reused and other patterns can be printed onto the paper, and the schematic illustration of the above process is shown in Fig. 4(e).

**Figure 5:**
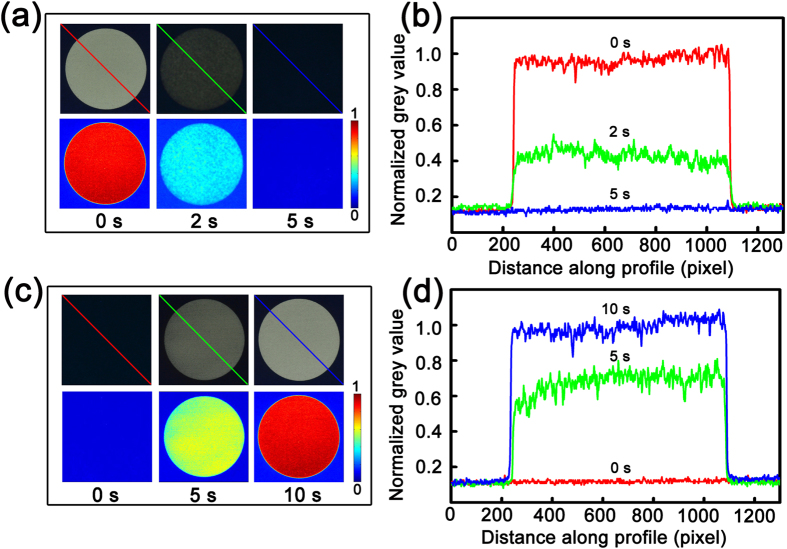
(**a**) The printed images and MATLAB treated patterns of three circles placed above vinegar vapor for 0 second, 2, and 5 seconds; (**b**) The intensity of the circles calculated with MATLAB along the diagonal line marked in (**a**); (**c**) The images and MATLAB treated patterns of the reprinted circles using ZnO NPs as ink after a neutralization process; (**d**) The intensity of the circles calculated with MATLAB along the diagonal line marked in (**c**).

The time that will take for the erasing and neutralizing process is an important issue of this route that should be concerned. To test the time for the erasing and the neutralizing process, three circles with a diameter of 3 cm have been printed onto a piece of paper using the ZnO NPs as ink, and then the paper is placed above the vinegar vapour for 0 second, 2 seconds, and 5 seconds, respectively. For intuitive observation, the printed patterns have been simulated using MATLAB, as shown in Fig. 5(a). Also, the normalized grey value along the diagonal of the three circles has been calculated with MATLAB and indicated in Fig. 5(b). The grey value of the cycle placed above vinegar vapour for 0 second has been set as unity. The value of the circle placed above vinegar vapour for 2 seconds falls to about 0.4, and that of the circle after being placed above vinegar vapour for 5 seconds is almost 0, indicating that the printed pattern can be erased completely within 5 seconds. Then the acid adsorbed paper is placed above soda vapour for 0 second, 5 seconds and 10 seconds, respectively, and another three circles are printed onto the paper using the ZnO NP solution as ink. The printed patterns are simulated with MATLAB as well, as shown in Fig. 5(c). The grey value along the diagonal of the printed circles in Fig. 5(c) has been calculated in the same process, as shown in Fig. 5(d). The grey value of the three printed circles is about 0, 0.6 and 1 correspondingly, which indicates that after 10 seconds neutralizing process in the ambient of soda vapour the paper can be reused again.

**Figure 6:**
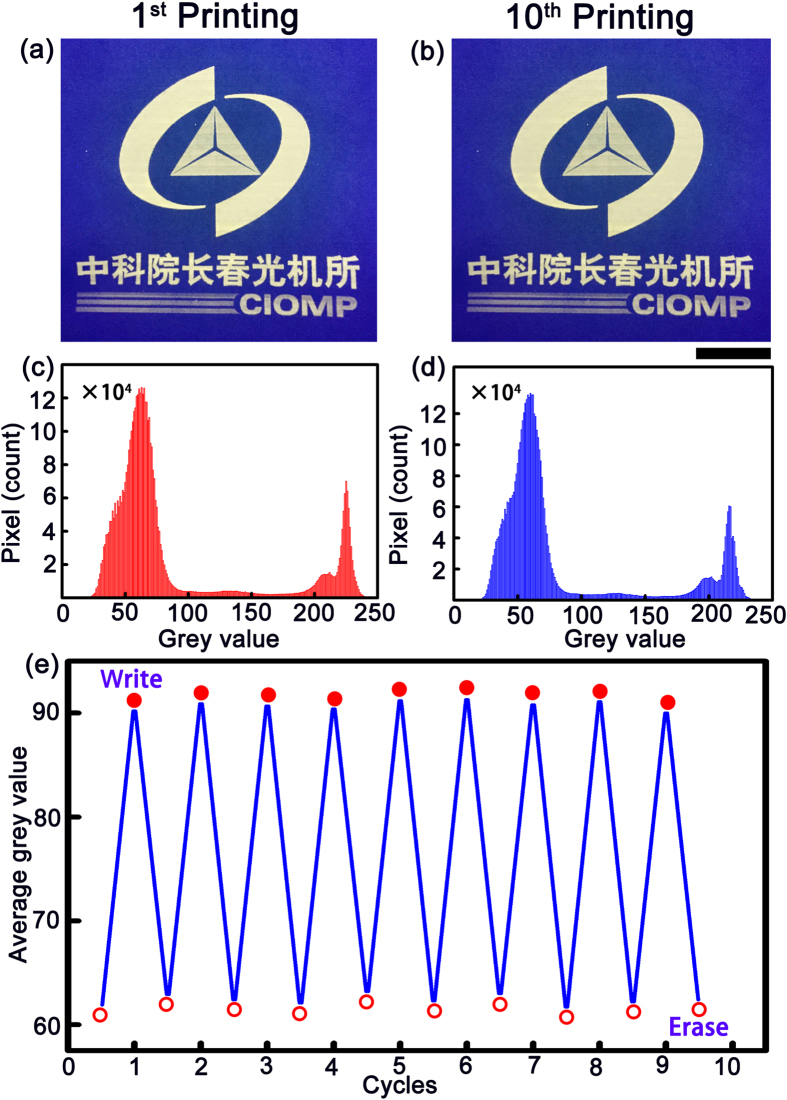
(**a**) The image printed onto a piece of paper for the 1^st^ cycle; (**b**) The image printed on the paper for the 10^th^ cycle; The grey value distribution of each pixel in the 1^st^ image (**c**) and that of the 10^th^ image (**d**); (**e**) A plot of the averaged grey values for the image in the ten cycles (Scale bar = 1 cm).

To test the quality of the rewritten patterns after several cycles, an image (The logo) has been employed as an example. Firstly, the image is printed onto a piece of paper with ZnO NP solution as ink, as shown in Fig. 6(a). Then the image is erased, and the paper is neutralized, and then the image is reprinted onto the paper again, the above process corresponds to one cycle. The images produced for the first cycle and the tenth cycle have been illustrated in Fig. 6(a) and (b). The grey value of each pixel of the first and tenth printed image has been calculated and shown in Fig. 6(c) and (d). It is obvious that the grey value distribution of the two patterns are similar, indicating that the quality of the pattern degrades little after ten rewriting cycles. The average grey value of the images produced in the ten cycles has been calculated with MATLAB, as shown in Fig. 6(e). It can be seen that the average grey value is almost constant for the ten sequentially printed patterns, and that for the erased sample is also very similar. The above results mean that the precision and resolution of the images produced in the above process degrades little after ten cycles, confirming the validity of the rewriting route demonstrated in this paper.

**Figure 7:**
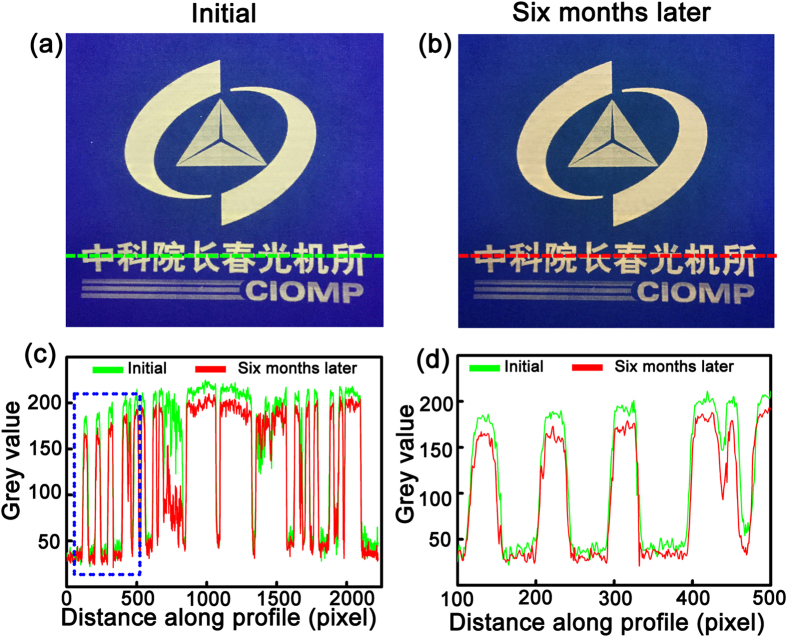
The image of the as-printed pattern (**a**) and that of the pattern after being stored in air ambient for six months (**b**); (**c**) The grey value along the lines marked in (**a**) and (**b**); (**d**) The magnified version of the rectangle marked in (**c**).

Storability is another important factor determines the usefulness of this rewriting route. To test the storability, the image produced using the ZnO NPs as fluorescence ink has been stored in ambient conditions, and the photo of the as-printed image and that of the image six months later have been listed in Fig. 7(a) and (b). The grey value along the lines marked in Fig. 7(a) and (b) has been calculated, as shown in Fig. 7(c). For clarifying, the area marked by the rectangle in Fig. 7(c) has been magnified and shown in Fig. 7(d). One can see that the shape of the grey value curves in these two patterns is almost the same although the grey values are slightly different, which indicates that the image produced in this route can be stored for a relatively long time, promising the usefulness of this rewritable route further.

## Conclusion

A simple route to rewriting on paper has been demonstrated employing ZnO NPs as fluorescent ink, and the quality of the image degrades little after ten cycles of usage; also the image produced in this route can be stored for several months. Considering the agents like vinegar, soda, and ZnO, used in this study are all cheap in cost and good eco-friendliness, the results reported in this communication may be of great significance in reducing the consumption of paper, thus help to abate the pollution caused in paper making process.

## Methods

### Materials

The reagents used in our experiments were zinc acetate dehydrate (Zn(Ac)_2_·2H_2_O), potassium hydroxide (KOH), (3-(2,3-epoxypropoxy)-propyl) trimethoxysilane (APTES), ethanol, vinegar, and sodium carbonate (soda). The vinegar and soda are of edible grade, and other chemicals regents are of analytical grade and used as received without further purification.

### Synthesis of water-soluble ZnO NPs

In this study, APTES was carried out during the growth process of ZnO NPs to prepare water-soluble ZnO NPs. The preparation procedure of water-soluble ZnO NPs is illustrated as follows: A 5.5 g (25 mmol) Zn(Ac)_2_·2H_2_O was dissolved in 150 ml ethanol solution, and the solution was refluxed at 80 °C for 2 hours under continuous stirring. Then 20 ml 1.75 M KOH ethanol solution was added into the Zn(Ac)_2_·2H_2_O ethanol solution for 30 minutes under continuous stirring at 0 °C until it became colorless and transparent. Finally, 1.5 ml deionized water and 400 μl PTES were added into ZnO NPs solution until it became turbid. After that, the solution was centrifuged (6000 rpm, 10 min) and the obtained precipitation was washed using ethanol for several times to remove the unreacted precursors. The washed precipitation was then dispersed in 75 ml deionized water for further applications.

### Characterizations

The structural properties of ZnO NPs were characterized by using a JEM-2010 transmission electron microscope and a Bruker D8 Discover (Germany) x-ray diffractometer. The fluorescence properties of the NPs were collected in a Hitachi F-7000 fluorescence spectrophotometer. The absorption spectra of the samples were measured in Shimadzu UV-3101PC spectrometer. The FTIR spectrum of NPs was obtained with a Bruker VERTEX-70 FTIR spectrometer.

## Additional Information

**How to cite this article**: Liu, K.-K. *et al*. Rewritable Painting Realized from Ambient-Sensitive Fluorescence of ZnO Nanoparticles. *Sci. Rep.* **7**, 42232; doi: 10.1038/srep42232 (2017).

**Publisher's note:** Springer Nature remains neutral with regard to jurisdictional claims in published maps and institutional affiliations.
